# YAP and 14-3-3γ are involved in HS-OA-induced growth inhibition of hepatocellular carcinoma cells: A novel mechanism for hydrogen sulfide releasing oleanolic acid

**DOI:** 10.18632/oncotarget.10663

**Published:** 2016-07-18

**Authors:** Guanglin Xu, Jing Wang, Fangfang Wu, Na Wang, Wenli Zhou, Qian Wang, Wang Pan, Guizhen Ao, Jiquan Yang

**Affiliations:** ^1^ College of Life Sciences, Nanjing Normal University, Nanjing, China; ^2^ Jiangsu Key Laboratory of 3D Printing Equipment and Manufacturing, Nanjing Normal University, Nanjing, China; ^3^ Jiangsu Key Laboratory for Molecular and Medical Biotechnology, College of Life Science, Nanjing Normal University, Nanjing, China; ^4^ Department of Medicinal Chemistry, School of Pharmacy, Soochew University, Jiangsu, China

**Keywords:** YAP, 14-3-3, COX-2, HCC, Hep G_2_

## Abstract

Hydrogen sulfide-releasing oleanolic acid (HS-OA) is an emerging novel class of compounds and consists of an oleanolic acid (OA) and a H_2_S-releasing moiety. Although it exhibits improved anti-inflammatory activity, its potency in human cancers has not been understood yet. In this study, we examined the effects of HS-OA on the growth of liver cancer cell lines and the underlying mechanisms.

HS-OA inhibited the growth of all four cancer cell lines studied, with potencies of 10- to 30-fold greater than that of its counterpart (OA). HS-OA induced significant apoptosis and decreased viability, clonogenic activity and migration of Hep G_2_ cells. Further studies showed that HS-OA resulted in the reduction of YAP expression and its downstream targets, CTGF and CYR 61, thus promoting cell apoptosis. In addition, HS-OA caused a decrease of 14-3-3γ expression, which led to Bad translocation to the mitochondria, ΔΨm loss, cytochrome c release, caspase activation and a recovery of 14-3-3γ reversed these effects induced by HS-OA.

These findings indicate that YAP and 14-3-3γ are involved in HS-OA's effects on liver cancer cells and identifying HS-OA as a potential new drug candidate for cancer therapy.

## INTRODUCTION

Hepatocellular carcinoma (HCC) is generally acknowledged as the sixth most prevalent cancer in the world and is currently the third most common cause of cancer death with a 5-year survival rate of 7%. Most HCC patients are diagnosed at a late stage when curative treatment options are not applicable and the majority of death is due to relentless disease recurrence [1]. This underlines the necessity to uncover new etiological mechanisms and develop more effective approaches including targeted therapy for the prevention and treatment of HCC.

The Hippo pathway has been initially found in *Drosophila* and plays an important role in organ-size control and organism homeostasis. This pathway is a highly conserved pathway in mammals and its core components include MST 1/2, Lats 1/2, Yes-associated protein (YAP) and its paralog, TAZ. YAP is the major downstream effector of the Hippo pathway. It functions as a transcriptional co-activator and interacts with TEA Domain (TEAD) DNA binding proteins to initiate the expression of target proteins, such as Survivin, CTGF, Jag1, and Cyr61 [2].

Recently, YAP has been found to be involved in liver events. YAP activation can override cell-cell contact inhibition and promote cellular growth [3], which result in malignant transformation of mammary cells and hepatocytes [4]. A transgenic mouse model demonstrated that YAP over-expression caused a marked increase in liver size and eventually the formation of liver tumor. Particularly, YAP activation has been detected in clinical liver cancers, including HCC, where Yap nuclear localization has been observed in ~60% of cases, and in hepatoblastoma (HB), where its nuclear localization is evident in ~70% of cases [5].

The 14-3-3 proteins, first identified in 1967, are a family of 28- to 33-kd acidic polypeptides with conserved sequences found in eukaryotic organisms. There are 7 isoforms (β, ε, γ, η, σ, τ / θ, and ζ) in humans and they function by forming homo or hetero dimers and binding to phosphorylated-serine/threonine motifs on their target proteins. Through modulation of their binding partners, 14-3-3s have been implicated to regulate a diverse number of cellular processes [6, 7].

Recent studies demonstrated that expression of 14-3-3γ could promote cell proliferation [8] and that 14-3-3 γ could be identified as one of the HCC-related biomarkers [9, 10]. These studies suggested that the 14-3-3γ isoform might play an important role in tumor development and cancer progression.

NSAIDs are a class of drugs with a common feature of inhibiting the activity of cyclooxygenase (COX) enzymes and are widely used to treat inflammatory disorders, including osteoarthritis and rheumatoid arthritis. However, the detrimental effects of NSAIDs (ulceration, bleeding in gastrointestinal tract) and adverse effects in the cardiovascular and renal systems limit their utility in clinic [11]. Recently, a new class of drugs has been developed that are at least as effective as conventional NSAIDs in reducing pain and inflammation, but exhibit much greater safety in the GI tract [12]. These compounds consist of a hydrogen sulfide (H_2_S)-releasing moiety. H_2_S is a gaseous mediator that is known to exert cytoprotective, anti-inflammatory and antioxidant actions [13, 14]. The HS-OA is a newly developed compound which conjugates a hydrogen sulfide (H_2_S)-releasing moiety and oleanic acid. HS-OA has been shown to have stronger anti-inflammatory activity than oleanolic acid with no significant injury in gastrointestinal tract [15]. However, there have been no reports describing the effects of HS-OA on the growth of any human hepatic cancer cell lines or in any *in vivo* animal models of liver cancer.

In the present study, we investigated the effects of HS-OA on malignant biological behaviors of HCC and evaluated the underlying mechanisms. Our results showed that a new mechanism was involved in the apoptosis induced by HS-OA. In this mechanism: HS-OA resulted in a reduced YAP expression and downstream effectors, CTGF and CYR 61, thereby promoting cell apoptosis. In addition, HS-OA decreased 14-3-3 γ expression. The cytosolic binding of 14-3-3 γ with p-Bad was suppressed and mitochondria translocation of Bad was increased. Then, the interaction of Bad with Bcl-2 in mitochondria was facilitated, which caused attenuation of mitochondria membrane stability, cytochrome c release to cytoplasma and activation of caspases. All these findings suggest that YAP and 14-3-3 γ are involved in the HS-OA-induced inhibition of hepatocellular carcinoma cell growth.

## RESULTS

### HS-OA inhibited the growth of hepatocellular carcinoma cell lines

**Figure 1:**
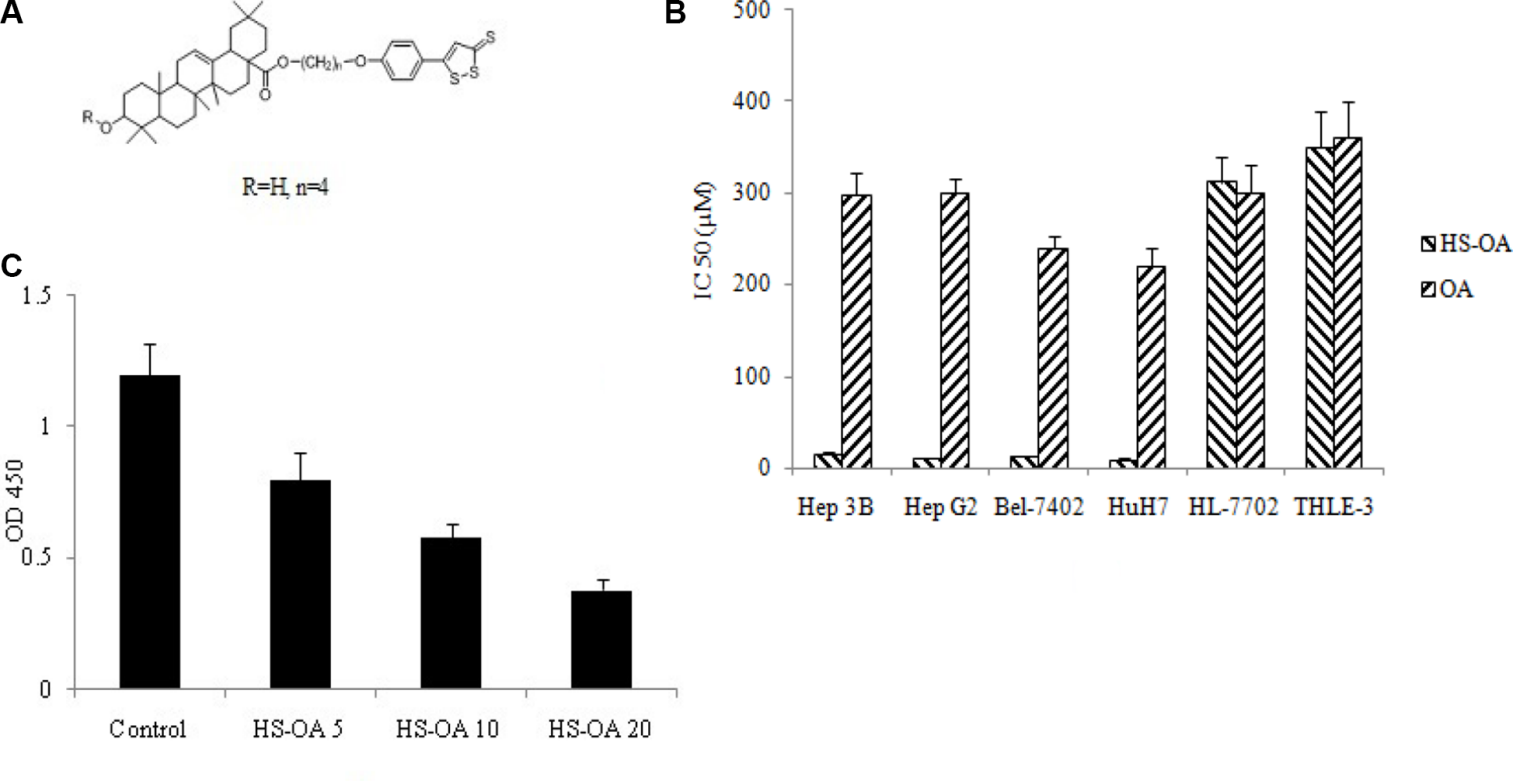
Effect of HS-OA on the growth of hepatocellular carcinoma cells and non-tumor cells Cells were treated with various concentrations of HS-OA and its counterpart OA. Cell numbers were determined at 48 h from which IC_50_ values were calculated. (**A**) Structure of HS-OA. (**B**) IC_50_ (μM) values for cell growth inhibition by HS-OA and OA in different cell lines. (**C**) Cell viability of Hep G_2_ on exposure to HS-OA. Cells were cultured in 96-well plates in the presence or absence of increasing concentrations of HS-OA (5, 10, 20 μM). Cell viability was then determined by using the WST-1 assay.

We investigated the effects of HS-OA (Figure 1A) and its respective parent compound (OA) on the growth properties of different hepatocellular carcinoma cells (Hep 3B, Hep G_2_, Bel-7402, HuH7 with high YAP expression) and non-tumor cells (HL-7702 and THLE-3 with low YAP expression). The HS-OA (48 h) was extremely effective in inhibiting the growth of HCC cell lines (Figure 1B.) and was much less potent in non-tumor cell lines (IC_50_ > 300 μM).

The growth inhibition by HS-OA versus its counterpart was very high in the cell lines studied. In a fold comparison study of the IC _50_ values (OA/HS-OA), HS-OA was more potent by 20-fold in Hep 3B cells, 30-fold more potent in Hep G_2_ cells, and 20-fold in Bel-7402 cells and 25-fold in HuH7 cells than OA. Such fold increases imply that the H_2_S-related structural modification of the parent molecule imparts an enhancement in potency. In order to determine the concentration of HS-OA for further studies, we performed a dose-response test on Hep G_2_ cells, in which HS-OA had the best inhibitory potency. As shown in Figure 1C, HS-OA at 20 μM showed an inhibition about 70%, and this effect is dose-dependent at concentrations of 5, 10, 20 μM. We chose 20 μM as the test concentration for further experiments.

### HS-OA led to reduction of YAP mRNA and protein expression and apoptosis

In order to investigate the detailed mechanisms of HS-OA in hepatocellular tumorigenesis, we used the Hep G_2_ cell line to explore what were the consequences when cells were treated with HS-OA. Because OA had no significant effects at the concentration of 20 μM, we focused on HS-OA's performance and the data regarding OA were not shown.

**Figure 2:**
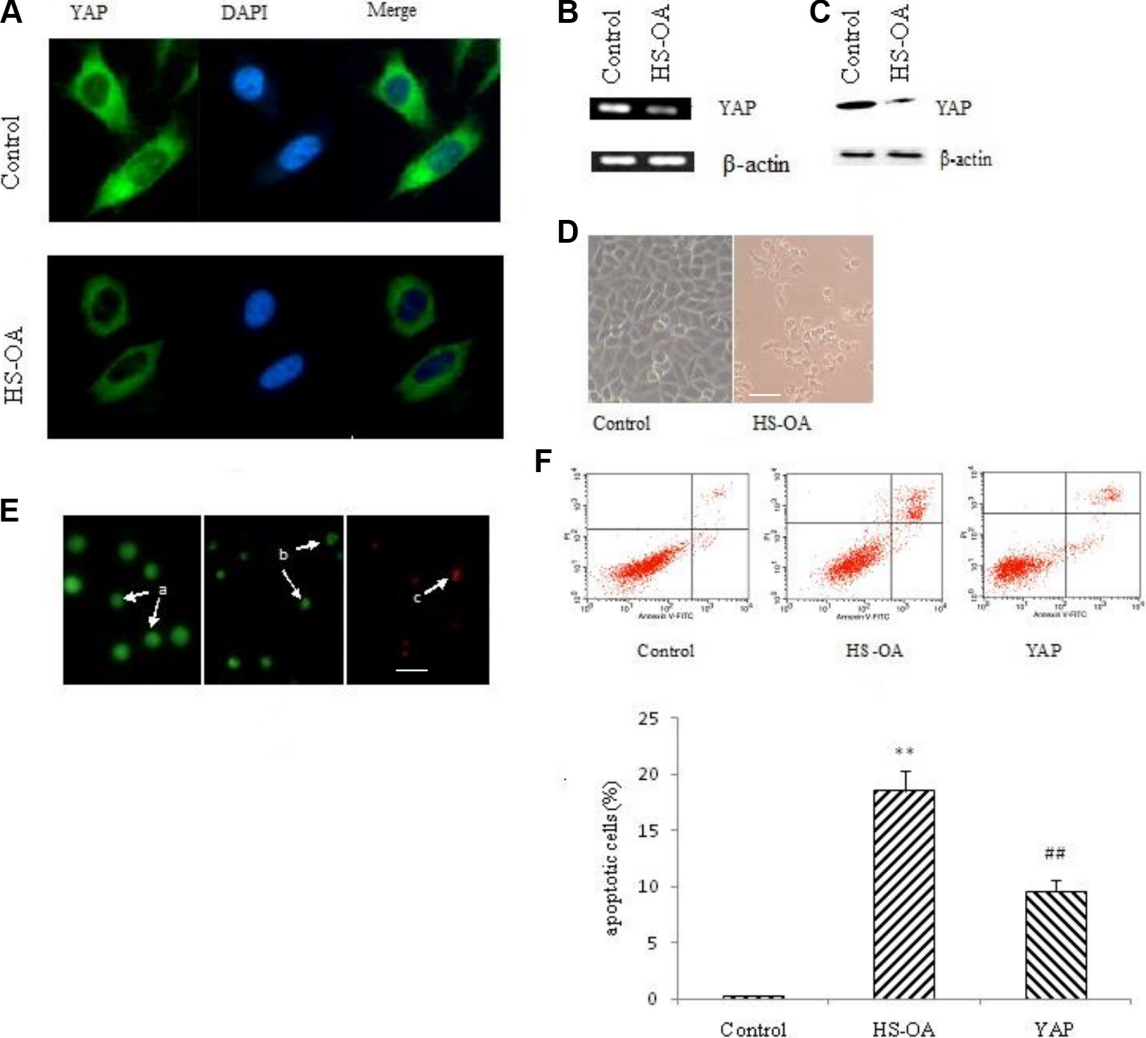
HS-OA (20 μM) decreased YAP expression and induced apoptosis Cells were treated with HS-OA or vehicle for 24 h in A, B, C and 48 h in D, E. (**A**) Expression of YAP and its subcellular localization examined by immunofluorescent confocal microscopy. Nuclei were stained with DAPI. (**B**) YAP mRNA expression determined by RT-PCR. (**C**) YAP protein expression detected by western blot. (**D**) Cell morphology was examined under an inverted light microscope. (**E**) Cells were seeded in 6-well plates (5 × 10^5^ cells/well) and incubated overnight before treated with vehicle or HS-OA. After 48 h of incubation, cells were collected and stained with 20 μL of aqueous AO/EB solution (100 μg/mL of AO and 100 μg/mL of EB in PBS) for 5 min. Cell morphology was observed under a fluorescent microscope. Arrow a: viable cell; b: early apoptotic cell; c: late apoptotic cell. Scale bar in D and E (for all pictures) = 20 μm; (**F**) Cells were treated with vehicle or HS-OA for 48 h, cells (1 × 10^5^) were collected and incubated with Annexin V and PI. The samples were analyzed by flow cytometry. Cells in early apoptosis are Annexin V positive and PI negative and cells in late apoptosis are positive for Annexin V and PI. **P < 0.01compared with control; ^##^*P* < 0.01 compared with HS-OA.

First, we evaluated the effect of HS-OA on YAP expression. The real-time quantitative PCR showed that HS-OA could markedly reduce the YAP mRNA level (Figure 2B). The Western blotting showed that treatment with HS-OA also inhibited the YAP protein expression (Figure 2C), which was consistent with the immunofluorescent staining. Namely, as shown in Figure 2A, HS-OA led to a weaker fluorescence than the untreated group.

Then, we observed morphological changes induced by HS-OA treatment. As shown in Figure 2D, cells treated with HS-OA tended to exhibit a round shape and became detached from the dish, which was accompanied by a reduced cell density. These changes seemed to be signs of typical hallmarks of apoptosis. To further investigate whether such changes were related to the induction of apoptosis, cells were analyzed using AO/EB staining by fluorescent microscopy.

As shown in Figure 2E, the control cells with intact DNA and nucleus gave a round and green nuclei with uniform chromatin and an intact cell membrane, while cells treated with HS-OA exhibited the characteristic changes of apoptosis, with cell shrinkage, nuclear condensation and fragmentation, formation of apoptotic bodies and gave condensed/fragmented green nuclei (early apoptosis) or were stained orange and red (late apoptosis). To further investigate HS-OA -induced apoptosis, we performed a quantitative flow cytometric analysis. As shown in Figure 2F, a small percentage of apoptotic cells were observed in control cells, whereas treatment with HS-OA resulted in a significant increase both in early and late apoptotic cells. In contrast, when YAP expression vector was transfected into the cells after HS-OA treatment, there was a significant decrease not only in early but also in late apoptotic cells. This result indicated that HS-OA was capable of inducing apoptosis whereas rescue of YAP expression was able to antagonize this effect.

### HS-OA inhibited cell viability, clonogenic activity and migration

**Figure 3:**
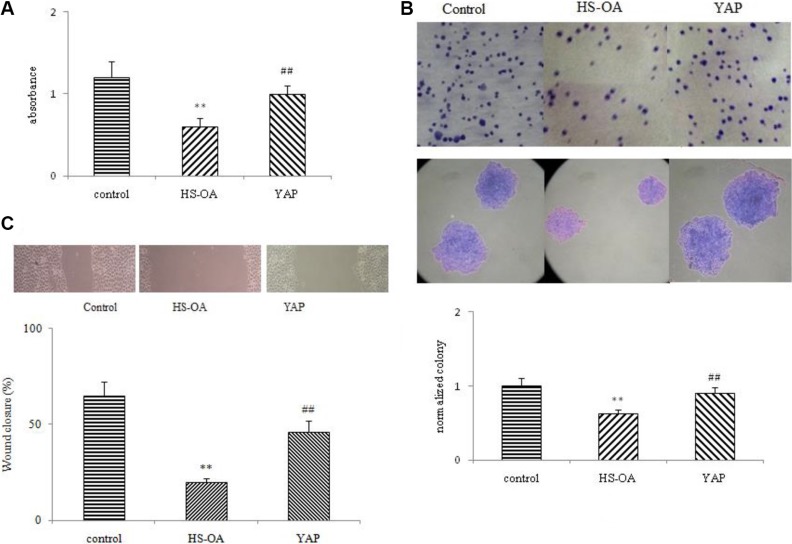
Effect of HS-OA on cell viability, clonogenic activity (20 μM) and migration (5 μM) Cells were treated with or without HS-OA for 24 h. (**A**) Cells were cultured in 96-well plates in the presence or absence of HS-OA. Cell viability was then determined by using the WST-1 assay. (**B**) Cells were seeded into 6-well plates and 9 days later, the colonies were stained with crystal violet, photographed (upper panel) and counted (lower panel). (**C**) Hep G_2_ cells were seeded into 6-well plates at 70–80% confluence. Cell migration was monitored by optical inspection for 24 h using a microscope and pictures were taken at 0 and 24 h (upper panel) and quantified. Each experiment was performed in triplicate, and the results represent the mean ± SEM of three experiments. **P < 0.01 compared with control; ^##^*P* < 0.01 compared with HS-OA.

To explore the behavior changes of HS-OA treatment in Hep G_2_ cells, cell viability, clonogenic activity and migration analysis were conducted. As shown in Figure 3A, WST-1 assay showed that after a 24-hour treatment, the viability was found to be reduced by ~50%. After a 48-hour treatment, this inhibitory effect became more potent (data not shown). As expected, when we rescued the YAP expression, the viability reversed to a significant higher level comparable to the control group.

In colony formation assay, cells treated with HS-OA showed much lower colony forming ability on plates than did the control cells. After seeding approximately the same cell number (200 cells each well), control cells grew readily and formed colonies on the bottom of the plates (Figure 3B). In contrast, HS-OA was sufficient to impair the clonogenic capability, leading to a drastic reduction in the number and size of colonies. Interestingly, when we rescued the YAP expression, the colony forming ability recovered to a similar level as the control cells indicating an important role of YAP in maintaining the clonogenic ability of test cells.

Finally, we addressed the question whether HS-OA interfered with Hep G_2_ cell migration. Enhanced migratory capacity is a particular feature of metastasizing HCC cells. For this purpose, a wounding assay was performed. In this assay cell proliferation was inhibited by addition of mitomycin C. As shown in Figure 3C, control Hep G_2_ cells showed most closure of a wound in a confluent monolayer after 24 h treatment, whereas HS-OA almost totally inhibited the wound closure. Even after 72 h there was only very limited number of tumor cells in the wound (data not shown). Taken together, HS-OA significantly inhibited cellular malignant biological behaviors of Hep G_2_ cells.

### Lats1 RNA interference increased the endogenous YAP expression and reversed the reduction of viability, clonogenic activity and migration induced by HS-OA in Hep G_2_ cells

**Figure 4:**
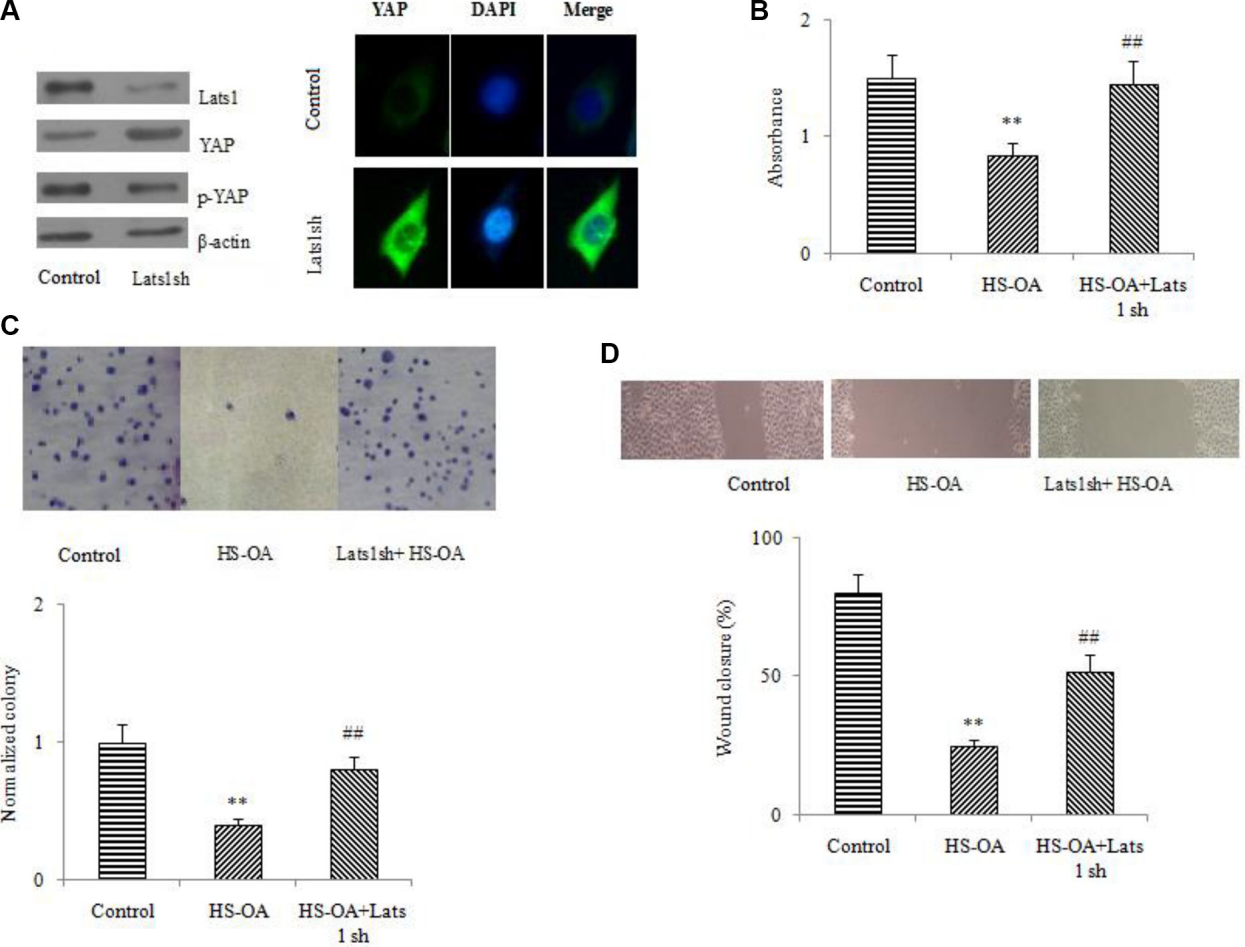
Effects of Lats1 disruption on the reduction of viability, clonogenic activity (20 μM) and migration (5 μM) induced by HS-OA (**A**) Regulation of Lats1, YAP and p-YAP by Lats 1 knockdown in Hep G_2_ cells. Protein levels of Lats1, YAP and p-YAP were determined by Western blot. (**B**) Hep G_2_ cells were transfected with sh RNA toward Lats 1 or non silencing sh RNA control. After transfection, these cells were treated with or without HS-OA for 24 h and then subjected to WST-1 assay. (**C**) Effect of Lats 1 sh RNA on HS-OA-induced colony formation. Cells were seeded into 6-well plates and 9 days later, the colonies were stained with crystal violet, photographed (upper panel) and counted (lower panel). (**D**) Effect of Lats 1 sh RNA on HS-OA-induced migration. Hep G_2_ cells were seeded into 6-well plates at 70–80% confluence. Cell migration was monitored by optical inspection for 24 h using a microscope and pictures were taken at 0 and 24 h (upper panel) and quantified (lower panel). **P < 0.01 compared with control; ^##^*P* < 0.01 compared with HS-OA.

Lats was identified as an important member of the Hippo pathway which could regulate organ size and cell proliferation. Lats was the upstream kinase of Hippo pathway, which was reported to be able to induce YAP phosphorylation and cytoplasmic location. Having shown that exogenous YAP introduction could antagonize the inhibitory effect of HS-OA on HCC growth, we further explored whether the endogenous YAP had similar effect on the growth of Hep G_2_ cells. We used the Lats1 sh RNA to silence Lats, which would result in an increase of endogenous YAP expression. Results showed that after treatment with Lats1sh RNA, the protein level of Lats 1 was significantly decreased (Figure 4A upper panel). Simultaneously, depletion of Lats1 significantly decreased the level of phosphorylated YAP and increased the level of YAP protein as well as its nuclear location (Figure 4A lower panel).

By WST-1 assay, the proliferation rate was significantly increased after transfecting with Lats1 sh RNA (Figure 4B). Consistent with the results of WST-1 assay, the knockdown of Lats 1 resulted in a significant increase in colony numbers as well as sizes (Figure 4C). To address the effect of Lats1 on Hep G_2_ cell migration, we performed wound-healing assay. As shown in Figure 4D, Lats 1-depleted Hep G_2_ cells showed significantly stronger migration ability compared with HS-OA treatment alone. These results indicated that Lats 1 could reverse the expression of YAP protein and the proliferation, clonogenic and migration ability in HS-OA-treated Hep G_2_ cells.

### Effect of HS-OA on apoptosis-related genes and downstream effectors

**Figure 5:**
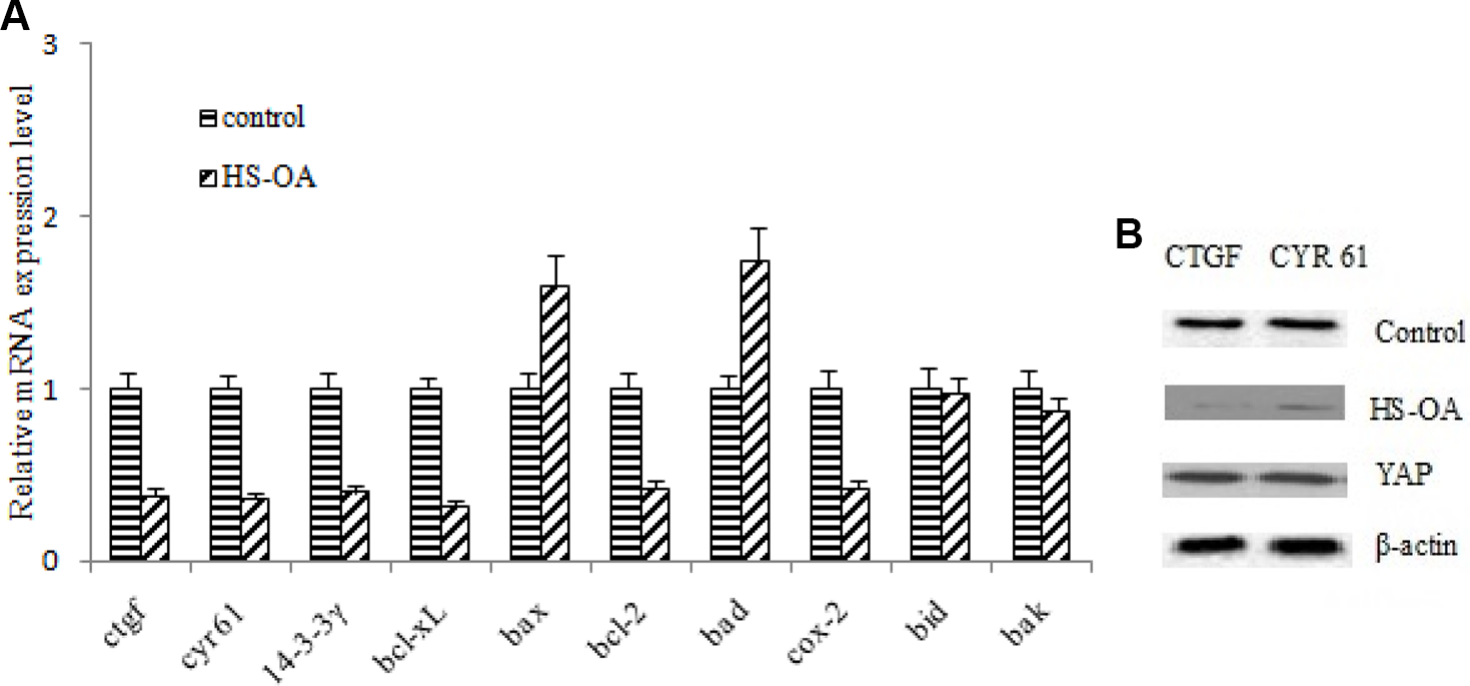
HS-OA (20 μM) altered the expression of apoptosis-related genes and downstream effectors (**A**) Cells were treated with HS-OA for 24 h and expressions of *ctgf, cyr 61, 14-3-3γ, bcl-xL, bax, bcl-2, bad, cox-2, bid, bak* were determined by quantitative RT-PCR and were normalized to expression of control. Experiments were repeated three times. (**B**) A decrease in expression of its target genes such as *ctgf, cyr 61* after incubation with HS-OA in Hep G_2_ cells for 24 h. A recovery of CTGF, CYR 61 expression was observed after YAP expression plasmids were transfected. *P < 0.05 compared with control.

To gain insights into molecular mechanisms of HS-OA -induced apoptosis, we performed mRNA expression analysis of related apoptotic genes after treatment with HS-OA. Among these genes, *ctgf, cyr 61, 14-3-3γ, cox-2, bcl-2, bcl-xL*, all of which are potent anti-apoptotic genes, were reduced. While *bax, bad,* which are potent pro-apoptotic genes were elevated (Figure 5A). These findings demonstrated that dysregulation of these proapoptotic and antiapoptotic genes was involved in HS-OA-induced apoptosis. In a further study, we also found that HS-OA led to decreased protein expressions of YAP target genes, such as *ctgf, cyr 61* in Hep G_2_ cells (Figure 5B). In addition, when YAP expression vector was applied after HS-OA treatment, the expression of CTGF, CYR61 recovered to a high level comparable to the control group.

### HS-OA reduced the 14-3-3γ expression and interaction of 14-3-3γ/ phospho-Bad and Bad/Bcl-2 (mitochondria)

**Figure 6:**
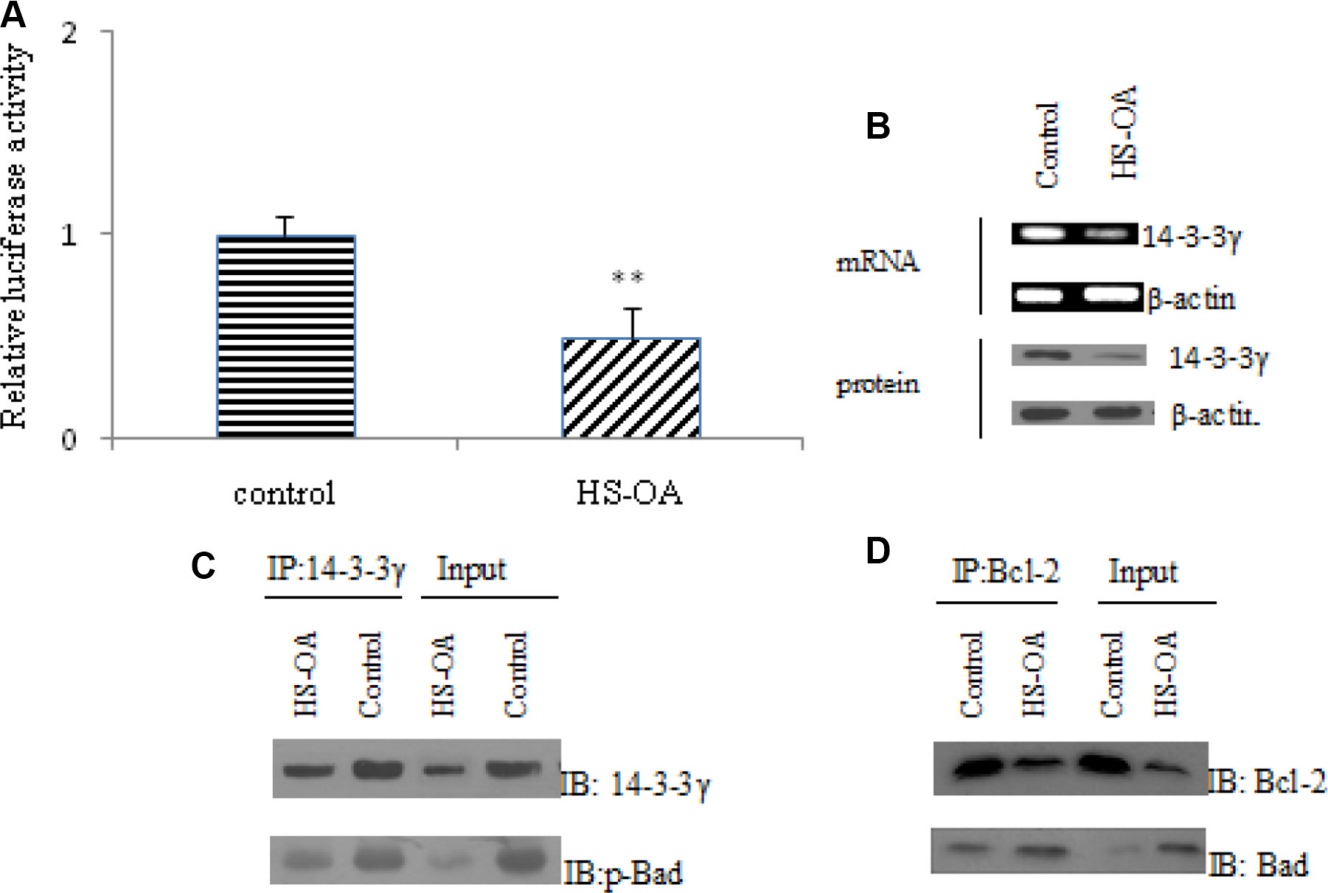
HS-OA (20 μM) influenced the 14-3-3γ expression and interaction of 14-3-3γ/ phospho-Bad and Bad/Bcl-2 (mitochondria) (**A**) 14-3-3 γ promoter activity was measured by a luciferase assay. The data were the average of three independent experiments. Cells were transfected with 14-3-3γ-luc reporter. (**B**) 14-3-3γ mRNA was measured by Q-RT-PCR and protein level was detected by western blot. (**C**) The interaction between 14-3-3γ and p-Bad in Hep G_2_ cells with or without HS-OA was examined by coimmunoprecipitation analysis. Anti-14-3-3γ antibody was used for immunoprecipitation (IP). The amount of p-Bad in the immunoprecipitates was detected by western blot (IB) with anti-p-Bad antibody. (**D**) The interaction between Bcl-2 and Bad in Hep G_2_ cells with or without HS-OA was examined by coimmunoprecipitation analysis. Anti-Bcl-2 antibody was used for immunoprecipitation (IP). The amount of Bad in the immunoprecipitates was detected by western blot (IB) with anti-Bad antibody. **P < 0.01 compared with control.

Of the altered apoptosis-related genes, 14-3-3γ attracted us most because it plays an essential role in the regulation of cell survival and apoptosis and influences other proteins such as Bad. So we next addressed the effect of HS-OA on 14-3-3γ expression and its functions. First, to determine whether HS-OA inhibits 14-3-3γ at the transcription level, we transfected Hep G_2_ with a human 14-3-3γ promoter construct. Results showed that HS-OA inhibited 14-3-3γ promoter activity (Figure 6A) as reflected by a significant decrease in luciferase reporter assay. HS-OA also induced inhibition of 14-3-3γ mRNA and protein expression (Figure 6B). Taken together, these results suggest that HS-OA suppresses 14-3-3γ at the transcriptional and translational level.

It is well known that 14-3-3 proteins are abundantly expressed adaptor proteins that interact with a vast number of binding partners to regulate their cellular localization and function. Of all of the proteins of the Bcl-2 family, Bad is the only member that has been demonstrated to bind to 14-3-3. Bad is phosphorylated on either Ser 112 or Ser136, and forms a complex with 14-3-3 in the cytosol. Then, to explore the effect of HS-OA on the physical interactions of 14-3-3γ with phospho-Bad (S112), co-immunoprecipitation was performed in Hep G_2_ cells. As shown in Figure 6C, phospho-Bad was coimmunoprecipitated with 14-3-3γ in control cells. When Hep G_2_ cells were treated with HS-OA, the precipitated phospho-Bad was markedly reduced with a concomitant reduced level of 14-3-3γ. These data indicated that treatment with HS-OA resulted in a low level of 14-3-3 γ and counteracted the mutual interaction of 14-3-3 γ with phospho-Bad. Finally, in a further study, CoIP was performed to verify the physical interactions of Bcl-2 with bad in mitochondria. As shown in Figure 6D, Bad was coimmunoprecipitated with Bcl-2 when cells were treated with HS-OA, indicating an increase of the formation of Bad/Bcl-2 heterodimer complex. These results indicated that HS-OA-induced reduction of 14-3-3γ suppressed its interaction with phospho-Bad, which drove Bad shift to the mitochondria and facilitated the interaction of Bad with Bcl-2, finally blocked the protective effect of Bcl-2 on the injury of mitochondria.

### 14-3-3 γ was involved in the subcellular location of Bad, cytochrom c release and Δψ_m_ loss and antagonizes the activation of Caspases

**Figure 7:**
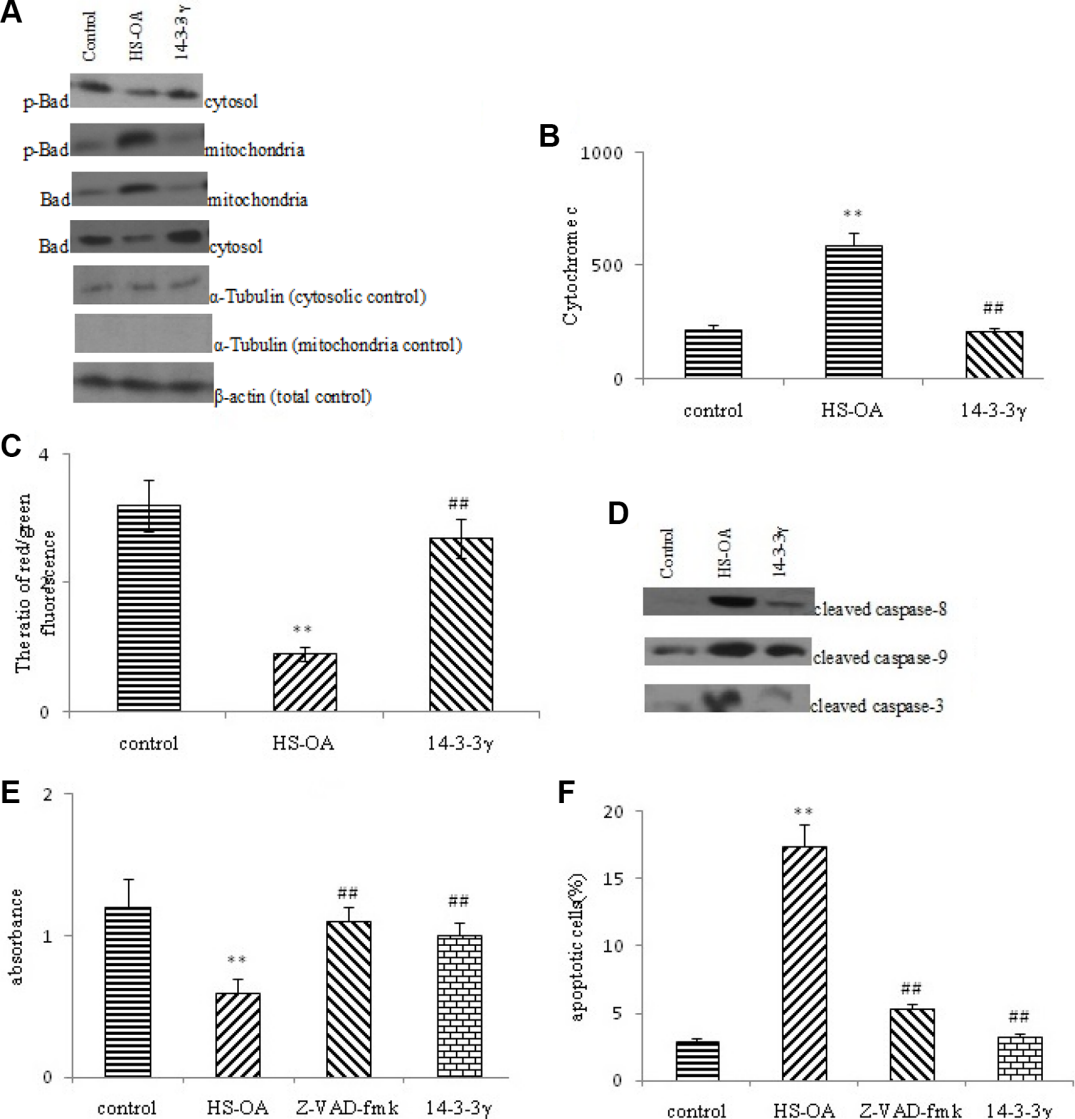
14-3-3 γ was involved in the subcellular location of Bad, cytochrom c release and Δψm loss and antagonizes the activation of caspases (**A**) Representative gel blots depicting the expression of p-Bad, Bad in the cytosolic and mitochondrial fractions of Hep G_2_ cells. Cells were transfected with p-14-3-3 γ or control vector, and the subcellular fractions (cytosolic and mitochondrial fractions) were obtained as described in the Materials and methods section. The total and sub-cellular extracts were immunoblotted using specific antibodies. α-tubulin and β-actin were used as the loading controls. (**B**) HS-OA -induced cytochrom c release to cytoplasm was measured by ELISA kits. (**C**) HS-OA -induced ΔΨm loss was measured by JC-1 staining and analyzed by flow cytometry. The quantitative data are presented as the ratio of the mean red fluorescence intensity to the mean green fluorescence intensity. Each bar represents the mean ± SEM of three independent experiments; (**D**) Effect of HS-OA on the protein levels of cleaved Caspase-8,9,3 in Hep G_2_ cells. (**E**) Effect of Caspase inhibitor z-VAD-fmk on HS-OA - induced cell viability in Hep G_2_ cells. (**F**) Evaluation of the percentage of apoptotic cells in HepG_2_ pre-treated for 2 h with the pan-Caspase inhibitor z-VAD-fmk (30 μM) and then co-treated with z-VAD-fmk (30 μM) and HS-OA for a further indicated time. **p < 0.01 compared with control group,^##^*p* < 0.01 compared with HS-OA group. HS-OA : 20 μM.

Cytosolic and mitochondrial fractions were prepared from untreated and HS-OA- treated or HS-OA plus 14-3-3 γ expressing vector treated cells to determine the subcellular locations of Bad and p-Bad and to monitor any changes associated with HS-OA. Our findings showed that p-Bad was mainly present in the cytosolic fractions from control cells and that Bad was present in both the mitochondrial and cytosolic fractions. The cytosolic level of p-Bad was increased and subcellular location of Bad underwent shifts from the cytosol to the mitochondria, which facilitated the interaction of Bad with Bcl-2 and blocked the protective effect of Bcl-2 on mitochondria (Figure 7A). Then cytochrome c was released to the cytosol after HS-OA treatment. Therefore, HS-OA-induced apoptosis was mainly caused by the mitochondrial translocation of Bad and subsequent release of cytochrome c from mitochondria. However, after 14-3-3 γ transfection, Bad shifted from the mitochondria to the cytosol and was sequestered in the cytosol, and the cytochrome c release to cytosol was significantly inhibited, indicating that HS-OA induced apoptosis via inhibition of 14-3-3γ and 14-3-3γ rescue may block apoptosis through a mitochondrial pathway triggered by the translocation of Bad (Figure 7B).

Because the mitochondria permeability transition sustained ΔΨm, we measured the ΔΨm using JC-1, which selectively enters the mitochondria and reversibly changes color from green to red when the ΔΨm increases. The ratio of the red to green fluorescent intensity thus reflects the ΔΨm. As shown in Figure 7C, HS-OA treatment induced a ΔΨm loss, and the ratio of the red to green fluorescent intensity was significantly decreased. In contrast, the transfection of p-14-3-3 γ expression vector inhibited the ΔΨm loss, as evidenced by an increase in the ratio.

The release of cytochrome c from mitochondria to the cytosol is believed to be an early event leading to the onset of apoptosis, which in turn activates the caspase family of proteases (such as Caspase-8, 9), finally the Caspase-3. This enzyme is, in fact, one of the most important executioners of the apoptotic process. To further investigate the mechanism and verify the effects of HS-OA on apoptosis, we measured the activities of Caspase-3, 8, 9 by using the antibodies that specially recognize these three enzymes (Figure 7D). Results showed that Caspase-8, 9, 3 were significantly activated after treatment with HS-OA when compared with untreated cells, which was evidenced by an increase in the intensity of cleaved-Caspase-8, 9, 3. To better understand the role of Caspase activation in HS-OA -induced apoptosis, Hep G_2_ cells were pre-treated for 2 h with the pan-caspase inhibitor z-VAD-fmk and then treated with HS-OA for a further 24 h. As shown in Figure 7F, treatment with HS-OA alone resulted in ~17% of apoptosis, whereas co-treatment with z-VAD-fmk resulted in ~5% apoptotic cells, suggesting that HS-OA -induced apoptosis occurred through a caspase-dependent pathway. In accordance with the above results, WST-1 assay showed that z-VAD-fmk could counteract the HS-OA -induced decrease in cell viability (Figure 7E). In addition, as expected, transfection of 14-3-3γ expression vector after HS-OA exhibited a similar effect as z-VAD-fmk not only in WST-1 assay but also in flow cytometric analysis.

### COX-2 and exogenous PGE_2_ reversed the changes of 14-3-3γ expression

**Figure 8:**
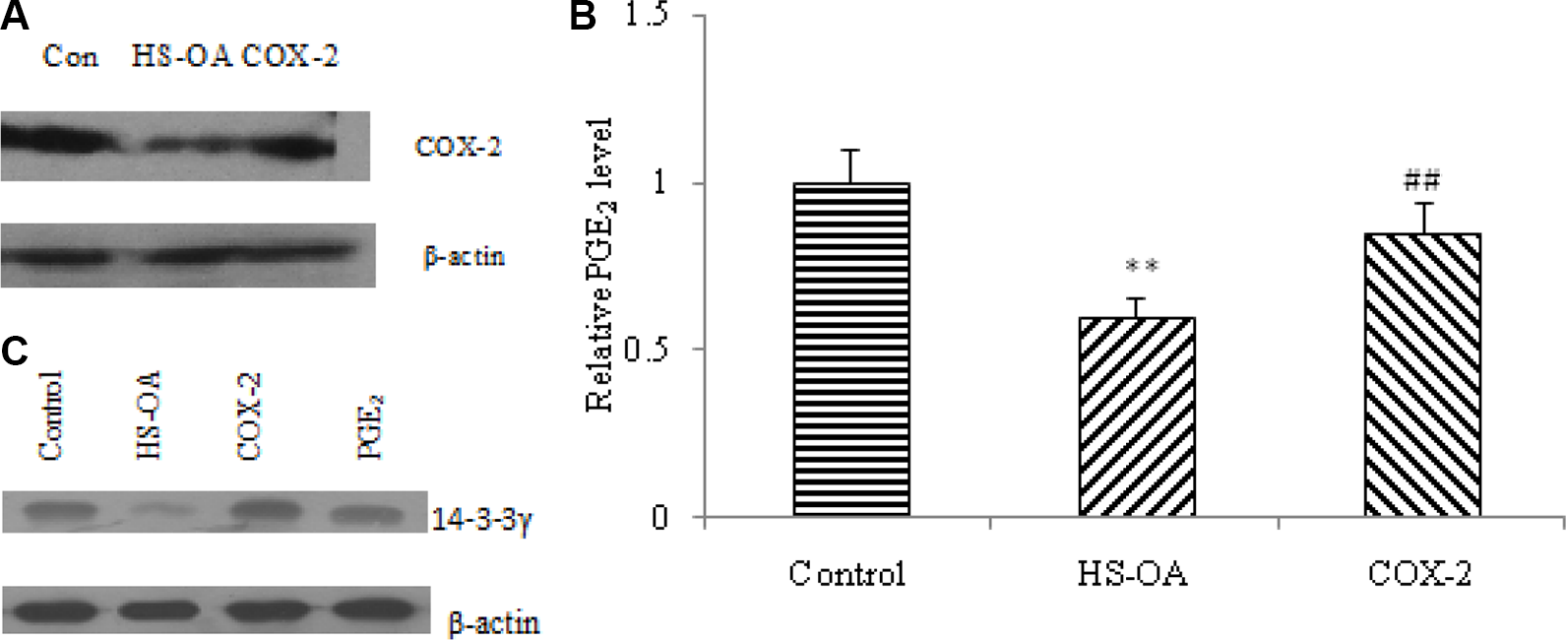
Effect of COX-2 expression vector transfection and exogenous PGE_2_ on HS-OA (20 μM)-induced 14-3-3γ protein expression (**A**) Cells transfected with or without COX-2 expressing vector were treated with or without HS-OA for 24 h, COX-2 expression was measured by western blot. (**B**) Cells transfected with or without COX-2 expressing vector were cultured in 96-well plates in the presence or absence of HS-OA. 24 h after incubation, supernatants were collected to measure PGE_2_ concentration by an enzyme immunoassay kit with a microplate reader according to the manufacturer's instructions. (**C**) Cells transfected with or without COX-2 expressing vector were treated with, without HS-OA or HS-OA in association with exogenous PGE_2_ for 24 h, 14-3-3γ expression was measured by western blot. **p < 0.01 compared with control group; ^##^*p* < 0.01 compared with HS-OA group.

Recently, cyclooxygenase-2 (COX-2), which is responsible for the production of prostaglandins, has been reported to be overexpressed in most human cancer cells. Its main metabolite, PGE_2_, can stimulate cellular migration, invasion, division and angiogenesis and inhibit apoptosis. Additionally, it has been shown that COX-2-derived PGE_2_ and PGI_2_ are capable of regulating 14-3-3 protein expressions. Considering HS-OA is a novel class of hydrogen sulfide-releasing compounds, which exerts a significant COX-2 inhibitory effect, we designed an experiment to test whether COX-2/PGE_2_ are involved in HS-OA-inhibited 14-3-3γ expression. Our results showed that HS-OA decreased the expression of COX-2 significantly as well as the level of PGE_2_ and COX-2-expressing vector could block this effect (Figure 8A, 8B). In a further study, we found that COX-2-expression vector was capable of antagonizing the decrease of 14-3-3γ expression induced by HS-OA. Furthermore, exogenous addition of PGE_2_ was able to reverse the HS-OA-induced decrease in 14-3-3γ expression (Figure 8B). These findings suggest that HS-OA -induced apoptosis was related to its ability of decreasing PGE_2_ level in Hep G_2_ culture medium and a COX-2-dependent mechanism is likely involved in this progress.

### Effect of 14-3-3 γ and COX-2 on tumor growth inhibition induced by HS-OA

**Figure 9:**
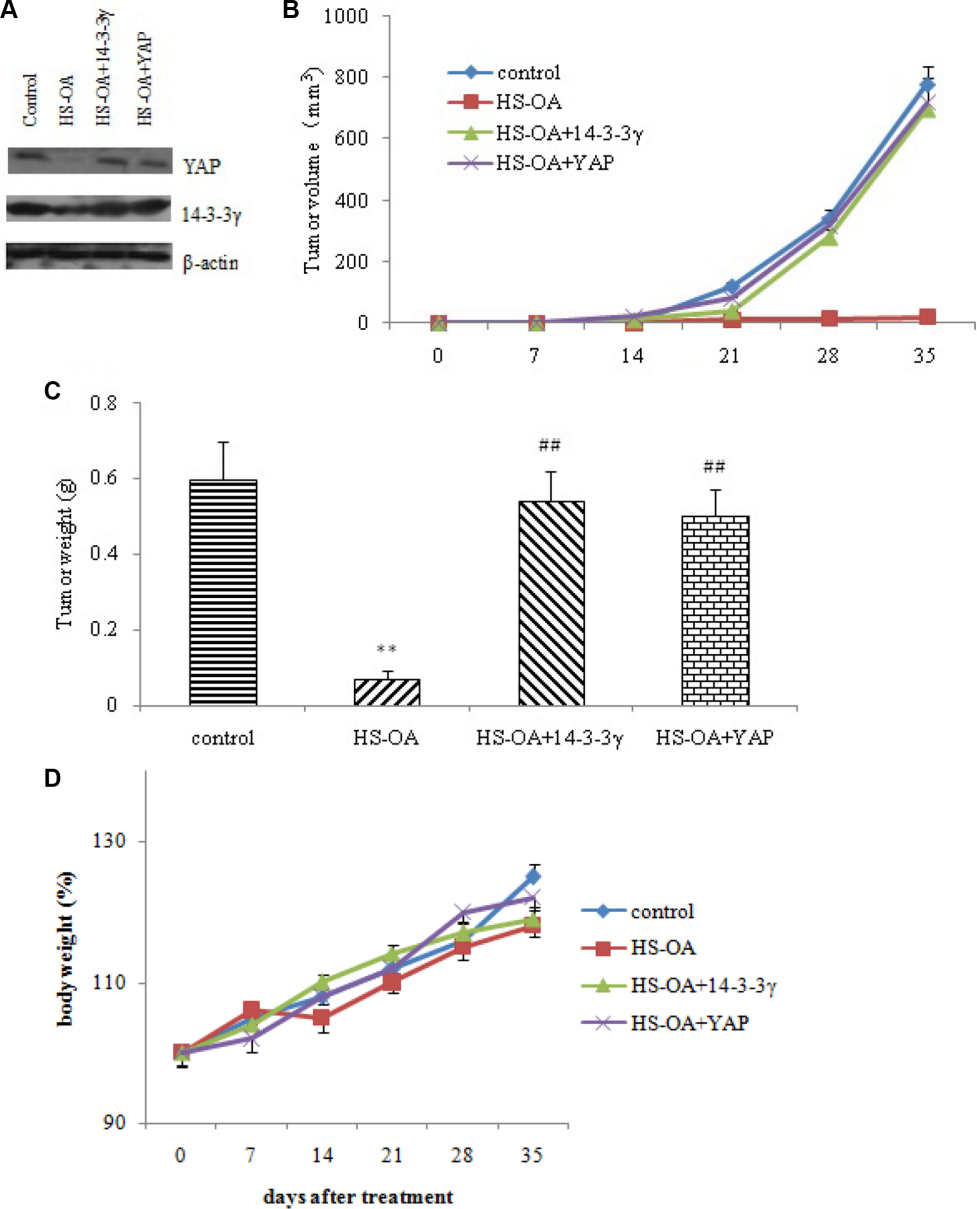
14-3-3γ and YAP rescues HS-OA -induced suppression of *in vivo* tumor growth Mice were randomly divided into four groups: control, HS-OA, HS-OA+14-3-3γ and HS-OA +YAP. HepG_2_ cells (5 × 10^6^) stably transfected with or without 14-3-3γ/YAP were subcutaneously (SC) injected into BALB/c mice. Mice were orally administered with HS-OA at a dose of 40 mg/kg/day in the indicated groups. Tumor size was measured every 7 days using a caliper, and tumor volume was calculated as 0.5 × L × W^2^, with L indicating length and W indicating width. Body weight was measured every 7 days until sacrifice. (**A**) YAP, 14-3-3γ expression in mice samples. Recovered expression of 14-3-3γ and YAP significantly increased *in vivo* tumor growth according to the results of calculated tumor volume (**B**) and tumor weight (**C**). (**D**) Body weight of mice in control, HS-OA, HS-OA+14-3-3γ and HS-OA +YAP groups at indicated times. (*n* = 6 each group) **p < 0.01 compared with control group,^##^*p* < 0.01 compared with HS-OA group.

The *in vivo* mouse model was used to test the effect of 14-3-3 γ on tumor growth inhibition caused by HS-OA. Control cells, 14-3-3 γ- and YAP vector-transfected cells were inoculated into mice respectively and then administered with HS-OA (40 mg/kg, p.o.). Tumor volumes and tumor weights were determined. From the day 21, tumor sizes were significantly smaller in mice with HS-OA than in control mice. 14-3-3γ expression plasmids significantly reversed the reduction of tumor volume and tumor weights as compared with HS-OA mice. This reversion was also observed in YAP-overexpressed animals (Figure 9B, 9C). These results suggest that the recovered 14-3-3 γ and YAP expression could antagonize the reduced tumor growth induced by HS-OA *in vivo*. In all of the tumor xenograft experiments done, there was no evidence of overt toxicity based on body weight and overall appearance of the treated animals (Figure 9D).

The western blot showed that HS-OA tumor tissues exhibited a low level of YAP and 14-3-3 γ protein. In contrast, HS-OA + 14-3-3 γ tumor tissues displayed an increased level of 14-3-3 γ (Figure 9A). Consistently, HS-OA + YAP tumor tissues displayed an increased level of YAP. These *in vivo* results are identical with the data obtained *in vitro* and support the idea that the recovery of 14-3-3 γ and YAP expressions can counteract the inhibitory effect of HS-OA on tumor growth.

## DISCUSSION

### HS-OA induced Hep G_2_ apoptosis

In the present study, we demonstrated that HS-OA inhibits the growth of HCC cell lines. This compound was more potent than its counterparts with enhanced potency ranging from 10 to 30 folds.

It is noteworthy that the potency of HS-OA in inhibiting the YAP-high expressing cell growth was more significant than in corresponding YAP-low expressing cells. This indicates that the inhibitory effect of HS-OA may be YAP dependent. Therefore, we next address this issue investigating the effect of HS-OA on the YAP expression in Hep G_2_ cells, which exhibit a high level of YAP. Results showed that YAP mRNA and protein level decreased to a low level after HS-OA treatment and cells displayed apoptotic morphological changes, indicating an occurrence of significant apoptosis. Moreover, HS-OA influenced malignant biological behaviors such as cell proliferation, migration and clonogenic activity. These results suggest that HS-OA has multiple effects on hepatocellular tumorigenesis.

Recently, YAP signaling has gained importance as a major regulator of organ size in various organs, including liver. In addition, YAP activation is found to be involved in numerous cancers [17, 18]. It is reported that overexpression of YAP has been found in approximately 60% of hepatocellular carcinomas [5]. Moreover the deregulation of the upstream members in the Hippo pathway has been reported in a broad range of different human carcinomas [19, 20]. However, the effects of Lats knockdown on the growth of Hep G_2_ and whether/how it affects the YAP expression have not been well characterized.

In this study, we found that Lats1 sh RNA treatment increased the level of YAP and facilitated its translocation to the nuclear. Further data showed that silence of Lats1 promoted the proliferation rate, colony numbers as well as sizes and migration of Hep G_2_ cells. These results revealed that upstream kinases of the Hippo pathway did inactivate the downstream key member, YAP, indicating that these core Hippo kinases retain a restraining activity on YAP function.

### HS-OA inhibited 14-3-3γ expression and resulted in concomitant downstream events

It was reported that 14-3-3 γ was found to be increased in HCC and overexpression of 14-3-3 γ contributed to liver tumorigenesis [9, 21]. Overexpression of 14-3-3 γ protein in human cancer cells resulted in abnormal cell proliferation and polyploidization [22]. Therefore, the 14-3-3γ protein is considered to be an interesting target linked to carcinogenesis.

In this study, we found that HS-OA inhibited 14-3-3γ promoter activity and resulted in decreases in 14-3-3γ mRNA and protein level. Furthermore, treatment with HS-OA reduced the mutual interaction between 14-3-3 γ and p-Bad and increased the formation of Bad/Bcl-2 heterodimer complexes in mitochondria, which subsequently promoted cytochrome c release from mitochondria to cytosol, activated Caspases and finally induced apoptosis. However, 14-3-3 γ expression vector transfection could drive Bad shift from the mitochondria to the cytosol and eventually alleviate HS-OA - induced apoptosis. Taken together, these results strongly suggest that 14-3-3 γ participates in the mechanisms by which HS-OA induces apoptosis.

### HS-OA decreased 14-3-3γ expression through a COX-2-dependent mechanism

COX-2 is primarily considered as an inducible enzyme, the expression of which is activated in response to cytokines, mitogens, endotoxin, and tumor promoters in a variety of cell types. The activation of COX-2 leads to the production of prostaglandin E_2_ and I_2_, which function in many aspects of pathophysiological processes, including pain and inflammation. However, recent evidence shows that COX-2 is also involved in most human cancers in which its main product, PGE_2_, stimulates cellular migration, invasion, division and angiogenesis and inhibits apoptosis [23]. It has been described in our work that COX-2 is highly expressed in Hep G_2_ cells [24]. In this study, our results revealed that HS-OA decreased the expression of COX-2 and the level of PGE_2_ in Hep G_2_ cells and either COX-2-expression vector or exogenous addition of PGE_2_ was capable of antagonizing the decrease of 14-3-3γ expression induced by HS-OA. These findings suggest that a COX-2-dependent mechanism is involved in this progress. But how COX-2 regulates the expression of 14-3-3γ?

It has been described that COX-2-derived prostaglandin I_2_ (PGI_2_) has been shown to activate PPARδ [18]. 14-3-3 transcription requires PPAR δ and is regulated by the level of PPAR δ [25], therefore HS-OA may induce apoptosis by blocking PGI_2_ production, thereby suppressing PPARδ activation and the subsequent inhibition of 14-3-3γ expression.

It has been accepted that PPARδ expression is regulated by the β-catenin-Tcf (T cell factor) transcriptional program and COX-2-derived prostaglandin E_2_ (PGE_2_) could increase β-catenin expression [26]. It is, therefore, possible that HS-OA may further suppress PPARδ expression through the β-catenin/Tcf pathway due to the inhibition of COX-2 and blockade of PGE_2_ production. Together, these data demonstrate that HS-OA decreases 14-3-3γ expression by a COX-2-dependent mechanism, i.e. inhibition of COX-2-derived PGE_2_ and PGI_2_ dual effects on PPAR δ activation.

### Complexity of YAP, COX-2 and 14-3-3γ in cancer

YAP, COX-2 and 14-3-3γ have all been found in a wide range of human cancers with high expressions. Nevertheless, how these components orchestrate to regulate the tumor growth and development? For instance, is there any mutual interaction between them? If yes, what are the detailed interaction sites and conformational changes? In addition, the regulation of one over another is monodirectional or bidirectional? Future investigation addressing these questions may provide new insights into the mechanism of tumorigenesis and possibly new therapeutic targets.

**Figure 10:**
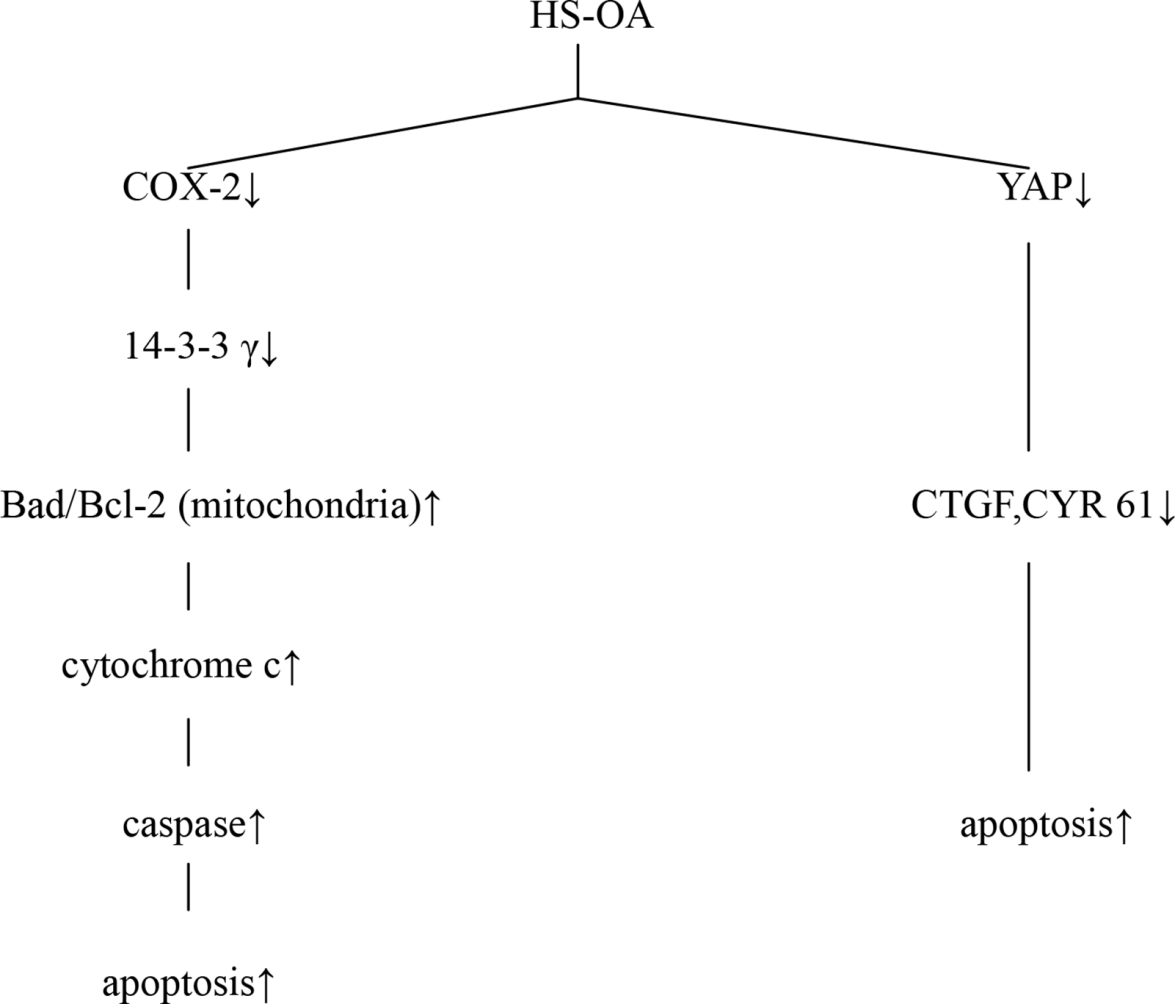
A schematic illustration of multiple biochemical processes via which HS-OA induces apoptosis

Taken together, our results suggest that HS-OA induces Hep G_2_ cell apoptosis as proposed in Figure 10. First, HS-OA suppresses COX-2 and thereby reducing 14-3-3 γ level and Bad sequestration. Second, HS-OA leads to excessive Bad translocation to the mitochondria with consequent loss of mitochondrial membrane potential, release of cytochrome c to cytoplasm resulting in caspase activation and ultimate apoptosis. Finally, HS-OA treatment results in reduction of YAP expression and subsequent decreases of target targets such as CTGF, CYR 61, therefore promoting cell apoptosis. These processes may be interrelated and mutually reinforcing.

In conclusion, our findings in this report provide new insights into HS-OA effects on HCC cells, identifying YAP and 14-3-3γ as novel modulators of these events and potentially provide new chemicals and targets for HCC cancer therapy.

## MATERIALS AND METHODS

### Chemicals and reagents

HS-OA was synthesized in our lab. PGE_2_ (Cayman Chemical, Ann Arbor, MI, USA); Dulbecco's modified eagle medium (DMEM) and fetal bovine serum (FBS) (Gibco BRL, USA); trypsin, acridine orange (AO), ethidium bromide (EB), propidium iodide (PI) and WST-1 (Roche, Mannheim, Germany); penicillin and streptomycin (Sunshine Biotechnology, Nanjing, China); antibodies to caspase-3,8,9, Bad, p-Bad, Bcl-2, p-YAP, Cyr 61, 14-3-3γ and horseradish peroxidase (HRP)-linked anti-rabbit IgG were obtained from Cell Signaling (Beverly, MA, USA). Antibodies to YAP, CTGF, CYR 61, Lats1, HRP-linked goat anti-mouse IgG were purchased from Santa Cruz Biotechnology (Santa Cruz, CA, USA). The pancaspase inhibitor z-VAD-fmk was from R&D Systems (Minneapolis, MN). Other agents were the highest quality available in market. Lats 1 sh RNA was obtained from Santa Cruz (USA).

### Cell lines

Hep 3B, Hep G_2_, Bel-7402, HuH7, THLE-3, HL-7702 were obtained from the cell bank of Shanghai Institute of Cell Biology (Shanghai, China) or ATCC. Cells were cultured in 75- or 150- cm^2^ flasks with Dulbecco's modified eagle medium (DMEM) supplemented with 10% fetal bovine serum (FBS), 100 U/mL penicillin, and 100 μg/mL streptomycin. Cells were incubated in a 5% CO_2_ incubator at 37°C.

### Cell viability assay

Cell viability during incubations of cultured hepatocytes was measured using the cell proliferation reagent WST-1. The method is based on the cleavage of tetrazolium salt WST-1 by mitochondrial dehydrogenases in viable cells to a water soluble formazan dye. Following treatment, cells were treated by the addition of 10 μL WST-1 stock solutions per 100 μL of medium. After 2 h of incubation, the plate was shaken for 1 min and absorbance was determined at 450 nm on a microplate reader (Bio-RAD, USA). A decrease in cell viability was indicated by a decrease in the amount of formazan dye (decrease in absorbance). The ratios of OA/HS-OA represent fold-enhancement in potency of the HS-OA over the parent compound OA.

### Plasmid construction and transfection

YAP expression plasmids were obtained from addgene. To create the pGL3-COX-2 and pGL3-14-3-3 γ (p-14-3-3 γ) expression plasmids, the genomic DNA from human BxPC-3 cells was PCR amplified with forward primers hanging *Not* I site and reverse primers hanging *Xho* I site, products were digested and gel purified followed by ligation with *Not* I/ *XhoI* digested pGL3 linearized vector. These products were ligated together with *Not*I/*Xho*I digested PGL3 empty vector followed by transformation. All the expression constructs were sequenced and confirmed at the Nanjing Normal University sequencing facility. All transfections were conducted in 24-well plates. Approximately, 1 × 10^3^ cells/well were seeded 24 h before transfection. Plasmids were transfected into cells using Lipofectamine reagent (Life Technologies, Inc.).

### Knockdown of Lats by sh RNA using lentivirus gene delivery system

Lats retroviral constructs targeting Lats were purchased from Santa Cruz Biotechnology. For sh RNA transfection experiments, cells were transfected with scramble and Lats shRNA according to the manufacture's protocol. The efficiency of knockdown was confirmed by Western blotting and quantitative PCR.

### Acridine orange (AO) and ethidium bromide (EB) staining

Cells were seeded in 6-well plates (5 × 10^5^ cells/well) and incubated overnight before treated with vehicle or HS-OA. After 48 h of incubation, cells were collected and stained with 20 μL of aqueous AO/EB solution (100 μg/mL of AO and 100 μg/mL of EB in PBS) for 5 min. Then the cells were observed under a fluorescent microscope.

### Flow cytometry analysis of cell apoptosis

Apoptotic cells were analyzed using BD Pharmingen (San Diego, CA, USA) apoptosis kit, in accordance with the manufacturer's instructions. Briefly, following the treatment of cells with vehicle or HS-OA for 48 h, cells (1 × 10^5^) were collected and incubated with Annexin V and PI for 15 min at room temperature in a light-protected area. The samples were immediately analyzed by flow cytometry. Cells in early apoptosis are Annexin V positive and PI negative; however, cells in late apoptosis are positive for Annexin V and PI. Subsequent analysis was performed with CellQuest software and the total number of apoptotic cells (both early and late apoptosis) was quantified.

### Reverse transcription and real-time quantitative PCR

RNA preparation and reverse transcriptase polymerase chain reaction (RT-PCR). Total cellular RNA was extracted from both the controlled and treated cells in 6-well plates using TRIzol according to the manufacturer's recommendations (Invitrogen, Carlsbad, CA). From each sample, 1 μg of RNA was reverse transcribed (RT) using MgCl_2_ 2 μL, 10 × RT Buffer 1 μL, RNase Free dH_2_O 3.75 μL, dNTP 1 μL, RNase Inhibitor 0.25 μL, AMV Reverse Transcriptase 0.5 μL, oligo dT-Adaptor 0.5 μL. PCR analyses were performed on aliquots of the cDNA preparations to detect cyr 61, CTGF, 14-3-3γ, COX-2, bax, bcl-2, Bcl-xl, bad, bid, bak and β-actin (as an internal standard). The reactions were carried out in 5 × PCR Buffer 10 μL, Takara ExTaq HS 0.25 μL, cDNA, 5′and 3′primers 0.5 μL. The amplification conditions and primers are listed in Supplementary Table 1. After amplification, PCR products were electrophoresed on 1.8% agarose gels and visualized by ethidium bromide staining and UV irradiat ion.

### Isolation of mitochondrial and cytosolic fractions

The cells were harvested and washed once with PBS. The cell pellets were isolated by centrifugation at 800 g for 5 min at 4°C. The cell pellets were resuspended in solution (20 mM HEPES-KOH pH 7.4, 220 mM mannitol, 70 mM sucrose, 1 mM EDTA and 0.5 mM PMSF) and incubated on ice for 10–15 min. Next, the resuspended solution was transferred to a glass homogenizer and homogenized for 20–30 strokes with a drill-fitted pestle. The homogenate was centrifuged at 800 g for 5 min at 4°C. The supernatant was collected and centrifuged at 10,000 g for 10 min at 4°C. The resultant supernatant was collected to obtain the cytosol fraction. The mitochondrial pellet was further resuspended in solution (20 mM HEPES-KOH pH 7.4, 220 mM mannitol, 70 mM sucrose, 1 mM EDTA, 0.5 mM PMSF) and centrifuged at 10,000 g for 10 min at 4°C to yield an enriched mitochondrial fraction.

### Detection of the mitochondrial membrane potential (ΔΨm)

The mitochondrial membrane potential was evaluated using a mitochondrial membrane potential assay kit with JC-1 (Beyotime Biotechnologies, Jiangsu, China). After treatment, the cells were harvested, washed with PBS, and incubated with culture medium (without FBS) containing JC- 1 (2 mΜ) for 30 min at 37°C in the dark. After the JC- 1 was removed, the cells were washed with PBS, harvested by trypsinization, and resuspended in PBS. The amount of JC-1 retained by 10,000 cells per sample was measured at 530 nm (green fluorescence) and 590 nm (red fluorescence) with a flow cytometer and analyzed using the Cell Quest Alias software. CCCP-treated samples were used for the standard compensation. The data are presented as the ratio of red to green signals (590 nm/530 nm).

### Analysis of cytochrome c release

The concentration of cytochrome c outside mitochondria was measured by ELISA (Enzo Life Sciences Inc., Farmingdale, NY). Briefly, at the end of incubation, mitochondrial and cytosolic fractions were isolated from treated cells as described above. Fractions were then subjected to the assay and values are expressed as nanogram per milligram of total protein from each fraction.

### Immunoprecipitation

The immunoprecipitation was done as previously described with modifications [16]. Briefly, cells were harvested with immunoprecipitation (IP) buffer containing 40 mM Tris–HCl pH 7.5, 50 mM NaCl, 50 mM NaF, 100 μMNa_3_VO_4_, 0.2% NP-40, 10 μg/mL aprotinin and 10 μg/mL leupeptin. Insoluble materials were removed by centrifugation at 10,000 × g at 4°C for 10 min and the resultant supernatants (500 μg protein) were incubated with primary antibody (5 μg) overnight at 4°C. Fifty microliters of protein A/G plus-agarose (Santa Cruz Biotechnology) was added and the complex was incubated at 4°C overnight. The beads were washed three times with high salt buffer (1 M Tris-HCl, pH 7.4, 0.50 M NaCl, and 1% NP-40) and twice with lysis buffer to eliminate non-specific binding. The immunoprecipitated complexes were released with 2 × sample buffer for Western analysis.

### Western blot analysis of protein expression

Protein was extracted from cultured HepG_2_ cells and western blotting was performed as described [24]. The cells, at the end of incubation, were scraped from flasks and lysed in a lysis buffer (100 mL: Tris (pH7. 5) 50 mM, NaCl 150 mM, EDTA 1mM, SDS 0.1(m/v), Triton×-100 1% (v/v), PMSF 1 mM, aprotinin 10 μg/mL, leupeptin 10 μg/mL, pepstain 10 μg/mL) Then the samples were boiled at 100°C for 5 min and centrifuged at 13,000 rpm for 2 min at 4°C. Protein extracts were run on 12% SDS-PAGE gels and transferred to PVDF membrane (Millipore, USA). Following this, the membrane was blocked with 5% non-fat dry milk in TBS-T buffer for 1 h at room temperature. After the blocking, the membranes were incubated with appropriate dilution ratio of the relative primary antibody overnight at 4°C. The membranes were incubated with secondary antibody for 4 h at room temperature and detected with ECL reagent (Millipore, USA).

### Immunofluorescence

Cells grown on degreased glass coverslips to 60–80% confluence in regular culture medium were fixed in methanol/acetic acid (3:1, v/v) for 30 min at 4°C and permeabilized with 0.1% Triton ×-100 in PBS for 5 min. These cells were then rinsed and blocked for 1h in 5% fetal bovine serum at room temperature. The cells were then incubated with anti-YAP monoclonal antibody (Santa Cruz Bio-technology) and diluted 1:200 in PBS at 4°C overnight. After washing in PBS, the cells were incubated with a secondary fluorescein isothiocyanate-conjugated antibody (1:200, Sigma) for 1h at room temperature. After washing in PBS, the cells were incubated with PBS solution containing 1% DAPI and kept in dark place for 10 min. After extensive washing, the coverslips were inverted onto glass slides using Mowiol (Calbiochem) as a mounting medium. The slides were observed with a fluorescent microscope.

### Colony formation assay

For clonogenic assay, cells were transfected with sh RNAs (LATS 1) for 48 h, and then the cells were seeded out in appropriate dilutions into 6-well plates (200 cells per well), followed by incubation at 37°C for 9 days. The cells were then washed with 1× PBS and fixed with 4% paraformaldehyde at room temperature for 20–30 min. After washing with 1× PBS again, the cells were stained with 0.1% crystal violet/PBS, and the colonies with 50 or more cells were counted. All experiments were carried out in triplicate.

### Wounding assay

Hep G_2_ cells were seeded into 6-well plates at 70–80% confluence. To prevent proliferation during wounding assay, cells were treated with 10 μg/mL mitomycin C (Sigma-Aldrich) for 3 h. The cell monolayer was then scratched in the middle with a plastic tip. Cell migration was monitored by optical inspection for 24 h using an Olympus microscope (Olympus, Hamburg, Germany) and pictures were taken at 0 and 24 h.

### Luciferase reporter analysis

Hep G_2_ cells were seeded in 6-well plates. After reaching about 70% confluence, the cells were transfected with pGL3-basic vector, luciferase reporter constructs containing the 14-3-3γ promoter region, then stably cotransfected with a Renila luciferase expression plasmid into cells using Lipofectamine reagent (Life Technologies, Inc.). After transfection, the medium was replaced by fresh normal growth medium, and the cells were incubated for 24 h. After starvation in serum-free medium for 20 h, the cells were harvested, and the luciferase activity was determined by using a Dual-luciferase Reporter Assay system (Promega) that already contains an internal control detectable simultaneously with the luciferase reporter gene and was measured with a luminometer.

### Mouse tumor model

The BALB/c mice (Bikai, Shanghai, China) were randomly divided into four groups: control, HS-OA, HS-OA+14-3-3γ and HS-OA +YAP. HepG_2_ cells (5 × 10^6^) stably transfected with or without 14-3-3γ/YAP were subcutaneously (SC) injected into BALB/c mice. Mice were orally administered with HS-OA at a dose of 40 mg/kg/day in the indicated groups. Tumor size was measured every 7 days using a caliper, and tumor volume was calculated as 0.5 × L × W^2^, with L indicating length and W indicating width. Body weight was measured every 7 days until sacrifice. Mice were euthanized at 35 days after injection and specimens from representative tumor tissue were cut with a razor blade and frozen for western blot analysis of YAP, 14-3-3 γ expression. All animals were housed in a conventional animal facility with 12 h light/dark cycles and fed ad libitum. The animal experiment protocol was in accordance with the Chinese government guidelines and approved by the institutional ethics committee.

### Statistical analysis

The values are expressed as the means ± SEM from independent experiments. The differences in the means between each group were tested by one-way ANOVA followed by Student–Newman–Keuls test (comparisons between multiple groups); *p* < 0.05 was considered statistically significant.

## SUPPLEMENTARY MATERIALS TABLES



## Abbreviations

HS-OA: Hydrogen sulfide-releasing oleanolic acid
OA: oleanolic acid
HB: hepatoblastoma
TEAD: TEA domain
CTGF: connective tissue growth factor
Jag1: gene encoding Jagged-1
Cyr61: gene encoding Cysteine rich 61
HCC: hepatocellular carcinoma
COX: cyclooxygenase
YAP: Yes-associated protein
AO: acridine orange
EB: ethidium bromide
PI: propidium iodide
prostaglandin E2: PGE2
prostaglandin I2: PGI2
PPAR δ: peroxisome proliferator activated receptor δ
NSAIDs: Nonsteroidal anti-inflammatory drugs
Macrophage stimulating protein: MST
Large tumor suppressor: Lats
Water soluble tetrazolium: WST-1.

## References

[1] Huo XY, Zhang Q, Angela ML, Tang CJ, Gong YL, Bian JM (2013). Overexpression of Yes-associated protein confers doxorubicin resistance in hepatocellullar carcinoma. Oncol Rep.

[2] Tao J, Calvisi DF, Ranganathan S, Cigliano A, Zhou L, Singh S (2014). Activation of β-Catenin and Yap1 in human hepatoblastoma and induction of hepatocarcinogenesis in mice. Gastroenterology.

[3] Zhao B, Wei X, Li W, Udan RS, Yang Q, Kim J (2007). Inactivation of YAP oncoprotein by the Hippo pathway is involved in cell contact inhibition and tissue growth control. Genes Dev.

[4] Overholtzer M, Zhang J, Smolen GA, Muir B, Li W, Sgroi DC (2006). Transforming properties of YAP, a candidate oncogene on the chromosome11q22 amplicon. Proc Natl Acad Sci USA.

[5] Zhou D, Conrad C, Xia F, Park JS, Payer B, Yin Y (2009). Mst1 and Mst2 maintain hepatocyte quiescence and suppress hepatocellular carcinoma development through inactivation of the Yap1 oncogene. Cancer Cell.

[6] Liou JY, Ghelani D, Yeh S, Yeh S, Wu KK (2007). Nonsteroidal anti-inflammatory drugs induce colorectal cancer cell apoptosis by suppressing 14-3-3 epsilon. Cancer Res.

[7] Liou JY, Wu CC, Chen BR, Chen BR, Yen LB, Wu KK (2008). Nonsteroidal anti-inflammatory drugs induced endothelial apoptosis by perturbing peroxisome proliferator-activated receptor–delta transcriptional pathway. Mol Pharmacol.

[8] Hong SW, Qi W, Brabant M, Bosco G, Martinez JD (2008). Human 14-3-3 gamma protein results in abnormal cell proliferation in the developing eye of Drosophila melanogaster. Cell Div.

[9] Lee IN, Chen CH, Sheu JC, Lee HS, Huang GT, Yu CY (2005). Identification of human hepatocellular carcinoma-related biomarkers by two-dimensional difference gel electrophoresis and mass spectrometry. J Proteome Res.

[10] Cho WC (2007). Contribution of oncoproteomics to cancer biomarker discovery. Mol Cancer.

[11] Winkelmayer WC, Waikar SS, Mogun H, Solomon DH (2008). Nonselective and cyclooxygenase-2-selective NSAIDs and acute kidney injury. Am J Med.

[12] Wallace JL (2007). Hydrogen sulfide-releasing anti-inflammatory drugs. Trends Pharmacol Sci.

[13] Kimura H, Shibuya N, Kimura Y (2012). Hydrogen sulfide is a signaling molecule and a cytoprotectant. Antioxid Redox Signal.

[14] Li L, Bhatia M, Zhu YZ, Zhu YC, Ramnath RD, Wang ZJ, Anuar FB, Whiteman M, Salto-Tellez M, Moore PK (2005). Hydrogen sulfide is a novel mediator of lipopolysaccharide-induced inflammation in the mouse. FASEB J.

[15] Qiu T, Shi FC, Cheng J, Xu GL, Sheng JJ, Zheng WJ, Ao GZ A new class of compounds, their preparation and application.

[16] Nagata K, Puls A, Futter C, Aspenstrom P, Schaefer E, Nakata T, Hirokawa N, Hall A (1998). The MAP kinase kinase kinase MLK2 co-localizes with activated JNK along microtubules and associates with kinesin superfamily motor KIF3. EMBO J.

[17] Johns R, Halder G (2014). The two faces of Hippo:targeting the Hippo pathway for regenerative medicine and cancer treatment. Nat Rev Drug Disco.

[18] Zhao B, Li L, Lei QY, Guan KL (2010). The Hippo-YAP pathway in organ size control and tumorigenesis: an updated version. Genes Dev.

[19] Jimenez-Velasco A, Roman-Gomez J, Agirre X, Barrios M, Navarro G, Vazquez I (2005). Down-regulation of the large tumor suppressor 2 (Lats2/KPM) gene is associated with poor prognosis in acute lymphoblastic leukemia. Leukemia.

[20] Chakraborty S, Khare S, Dorairaj SK, Prabhakaran VC, Prakash DR, Kumar A (2007). Identification of genes associated with tumorigenesis of retinoblastoma by microarray analysis. Genomics.

[21] Morrison DK (2009). The 14-3-3 proteins: integrators of diverse signaling cues that impact cell fate and cancer development. Trends Cell Biol.

[22] Qi W, Liu X, Chen W, Li Q, Martinez JD (2007). Overexpression of 14-3-3gamma causes polyploidization in H322 lung cancer cells. Mol Carcinogen.

[23] Alexander G, Helena JM, Amy EM, Heather RR, Ann CW, Christos P, Abderrahmane K (2009). The COX-2/PGE_2_pathway: key roles in the hallmarks of cancer and adaptation to the tumor microenvironment. Carcinogenesis.

[24] Cheng J, Du YF, Xiao ZY, Pan LL, Li W, Huan L (2014). Growth inhibitory effect of KYKZL-1 on Hep G_2_ cells via inhibition of AA metabolites and caspase-3 pathway and cell cycle arrest. Toxicol Appl Pharmacol.

[25] Liou JY, Dipak G, Sam Y, Wu KK (2007). Nonsteroidal anti-inflammatory drugs induce colorectal cancer cell apoptosis by suppressing 14-3-3ε. Cancer Res.

[26] Castellone MD, Teramoto H, Williams BO, Druey KM, Gutkind JS (2005). Prostaglandin E2 promotes colon cancer cell growth through a Gs-axin-beta-catenin signaling axis. Science.

