# Supplementary material for: YAP and 14-3-3γ are involved in HS-OA-induced growth inhibition of hepatocellular carcinoma cells: A novel mechanism for hydrogen sulfide releasing oleanolic acid
Source: Oncotarget. 2016 Jul 18;7(32):52150–65. doi: 10.18632/oncotarget.10663 (PMC5239541; doi:10.18632/oncotarget.10663)
Supplement: Supplementary file 1 [file oncotarget-07-52150-s001.pdf]

YAP and 14-3-3γ are involved in HS-OA-induced growth inhibition of hepatocellular carcinoma cells: A novel mechanism for hydrogen sulfide releasing oleanolic acid

Supplementary Materials

Supplementary Table S1: The list of primers and conditions used in RT-PCR

Name	Primers	Conditions	Number of cycles
CTGF	F5′-TCTCCAACCTC TCCTA CTAC- 3′;<br>R5′-GCACGTAGTC TTCGATCACT-3′;	95°C for 15 s, 60°C for 1 min, 72°C for 1min	40
Cyr 61	F5 ′-CCCTTCCGAAGTTTCTGGCAGC- 3′<br>R5′-GGCTGTCAGAGCCTCGTGGCTT-3 ′	95°C for 15 s, 60°C for 1 min, 72°C for 1 min	40
14-3-3γ	F5′–CGTGCGTACCG GGAG AAG-3′;<br>R5′–TCCAGCAGGCTCAG CACA-3′;	94°C for 1min , 60°C for 1 min, 72°Cfor 2 min	35
Bcl-xL	F5′-ACCCATCCTGGCACCTGGCA-3′;<br>R5′-GGATCCAAGGCTCTAGGTGG-3′;	94°C for 30 s, 50°C for 30 s, 72°C for 1 min	35
bax	F5′-AAAGCTAGCGAGTGTCTCAAGCGC-3′<br>R5′-TCCCGCCACAAAGATGGTCACG-3′	94°C for 1 min, 60°C for 1 min, 72°C for 1 min	35
bcl-2	F5′-CGACGACTTCTCCCGCCGCTACCGC-3′<br>R5′-CCGCATGCTGGGGCCGTACAGTTC C-3′	94°C for 30 s, 50°C for 30 s, 72°C for 1 min	38
bad	F5′-TTTCTCGAGATGTTCCAGATCCCAGAGT TTGGG-3′,<br>R5′-TTTGAGTTCCTGGGAGGGGGCGGAGCT GTC-3′	94°C for 30 s, 50°C for 30 s, 72°C for 1 min	35
COX-2	F5′- TGA AAC CCA CTC CAA ACA CAC AG-3′<br>R5′- TCA TCA GGC ACA GGA GGA AG-3′	94°C for 30 s, 50°C for 30 s, 72°C for 2 min	35
bid	F5′-TTTGTTTGTGTGCTTCTGAGC-3′<br>R5′- C ATTCTGTTGCCACCTTTCGG-3′	94°C for 30 s, 50°C for 30 s, 72°C for 1 min	35
bak	F5′-GCTCTTCCTTTGTTCATCTCC-3′<br>R5′- CATCTGGCTCGGGGTTACTGC-3′	94°C for 30 s, 50°C for 30 s, 72°C for 1 min	35
β-actin	F 5′-TCATGAAGTGTGACGTTGAC AT C C GT-3′;<br>R5′-CCTAGAAGCATTTGCGGTGCACGATG-3′	94°C for 1 min, 60°C for 1 min, 72°C for 1 min	38
